# The role of asymmetrical prominent veins sign in early neurological deterioration of acute ischemic stroke patients

**DOI:** 10.3389/fneur.2022.860824

**Published:** 2022-08-15

**Authors:** Kuankuan Huang, Jianfang Liu, Wenwei Yun, Yin Cao, Min Zhang

**Affiliations:** ^1^First People's Hospital in Guangyuan, Guangyuan, China; ^2^Department of Neurology, Changzhou No. 2 People's Hospital Affiliated to Nanjing Medical University, Changzhou, China

**Keywords:** early neurological deterioration, asymmetrical prominent veins sign, ischemic stroke, white matter hyperintensities, middle cerebral artery (MCA)

## Abstract

**Background and purpose:**

Asymmetrical prominent veins sign (APVS) often appears on susceptibility-weighted angiography (SWAN) images in patients with acute stroke. Early neurological deterioration (END) is highly correlated with survival prognosis in patients with ischemic stroke. This study sought to explore the relationship between APVS and END in patients with acute stroke.

**Methods:**

The subjects retrospectively enrolled in this study were patients with acute ischemic stroke in the middle cerebral artery supply area. All patients underwent head MRI, including the SWAN sequence, within 7 days of stroke symptom onset. END was defined as clinical deterioration or recurrence within 72 h after ischemic stroke. The volume of infarction on diffusion-weighted imaging was measured. Univariate and multivariate analyses were used to analyze the relationship between APVS and END. Spearman correlation between APVS grades and infarct volume, white matter hyperintensity (WMH) volume, and offending vessel were also analyzed.

**Results:**

A total of 157 patients with middle cerebral artery infarct between September 2018 and April 2020 were included in the study. APVS appeared on MRI in 84 of 157 patients, and 34 of 157 patients were diagnosed with END. In patients with END, the proportion of severe APVS was higher than in patients without END (*P* = 0.001, *x*^2^ = 14.659). Patients with END were older and had a larger volume of infarct and WMH than patients without END (all *P* < 0.05). After adjustments were made for related risk factors of END, the severity of APVS was still related to END (OR = 2.56, 95% CI, 1.38–4.75; *P* for trend = 0.003). Spearman correlation showed that APVS grades were positively related to infarct volume (*r* = 0.289, *P* < 0.001) and 3-month modified Rankin Scale score (*r* = 0.203, *P* = 0.011) and negatively related to offending vessels (*r* = −0.170, *P* = 0.034).

**Conclusion:**

APVS may be an important predictor of END in patients with acute ischemic stroke.

## Introduction

Susceptibility-weighted angiography (SWAN), which is analogous to susceptibility-weighted imaging (SWI), is an imaging technique that identifies differences in magnetic sensitivity caused by unevenness of the local magnetic field (such as with blood or iron) in tissues. This technique can effectively display veins, blood metabolites, and iron deposits in patients with cerebrovascular disease. Compared with conventional MRI sequences, SWAN can more sensitively display cerebral venules, microbleeds, and iron deposition (1, 2).

The imaging of small veins with SWAN is based on the principle of blood oxygen level. On SWAN, patients with cerebrovascular disease may demonstrate the asymmetrical prominent veins sign (APVS), which refers to veins that are wider and longer in one hemisphere of the brain than in the other. APVS can be categorized based on location, with patients demonstrating the asymmetrical cortical veins sign (ACVS) or the asymmetrical medullary veins sign (AMVS) (3).

Deoxyhemoglobin is used as an endogenous contrast agent to display the structure of intracranial veins. Therefore, the degree of vein development in SWAN is mainly dependent on the amount of deoxyhemoglobin in venous blood (4). APVS is usually not present in healthy patients but is often present in patients who have experienced an ischemic stroke (5, 6).

The appearance of APVS is pathophysiologically complex. In the ischemic penumbra of patients with acute ischemic stroke, the brain tissue is in a state of poor perfusion. This results in increased tissue oxygen uptake increased concentration of venous deoxyhemoglobin and increased magnetic sensitivity of veins.

The clinical significance of APVS remains controversial. Some researchers hold the view that APVS in the infarct zone may represent the ischemic penumbra, as the oxygen uptake index in the penumbra zone is elevated, which is considered collateral branches of the ischemic penumbra. One study found that the presence of APVS is related to the early improvement of cerebral infarction (7). In a study of patients undergoing thrombolysis, those with AMVS identified on MRI before thrombolysis had better early reperfusion (8), which led researchers to conclude that the presence of APVS is associated with collateral circulation and is a good predictor of prognosis in patients with stroke (9, 10). However, other studies in patients with stroke who did not undergo thrombolysis demonstrated conflicting results, showing that the presence of APVS indicated poor arterial supply and was associated with adverse outcomes (6, 11). These conflicting findings obscure the clinical significance of APVS in patients with stroke. In particular, research has not yet clearly demonstrated the potential association between APVS and the presence of early neurological deterioration (END), which is a predictor of short-term and long-term prognosis and occurs in 10–40% of patients with ischemic stroke (12, 13).

The purpose of this study, therefore, was to explore the clinical role of APVS in cerebral infarction. In particular, we sought to determine whether a relationship exists between APVS and END in patients with acute stroke.

## Methods

### Study patients

**Figure 1:**
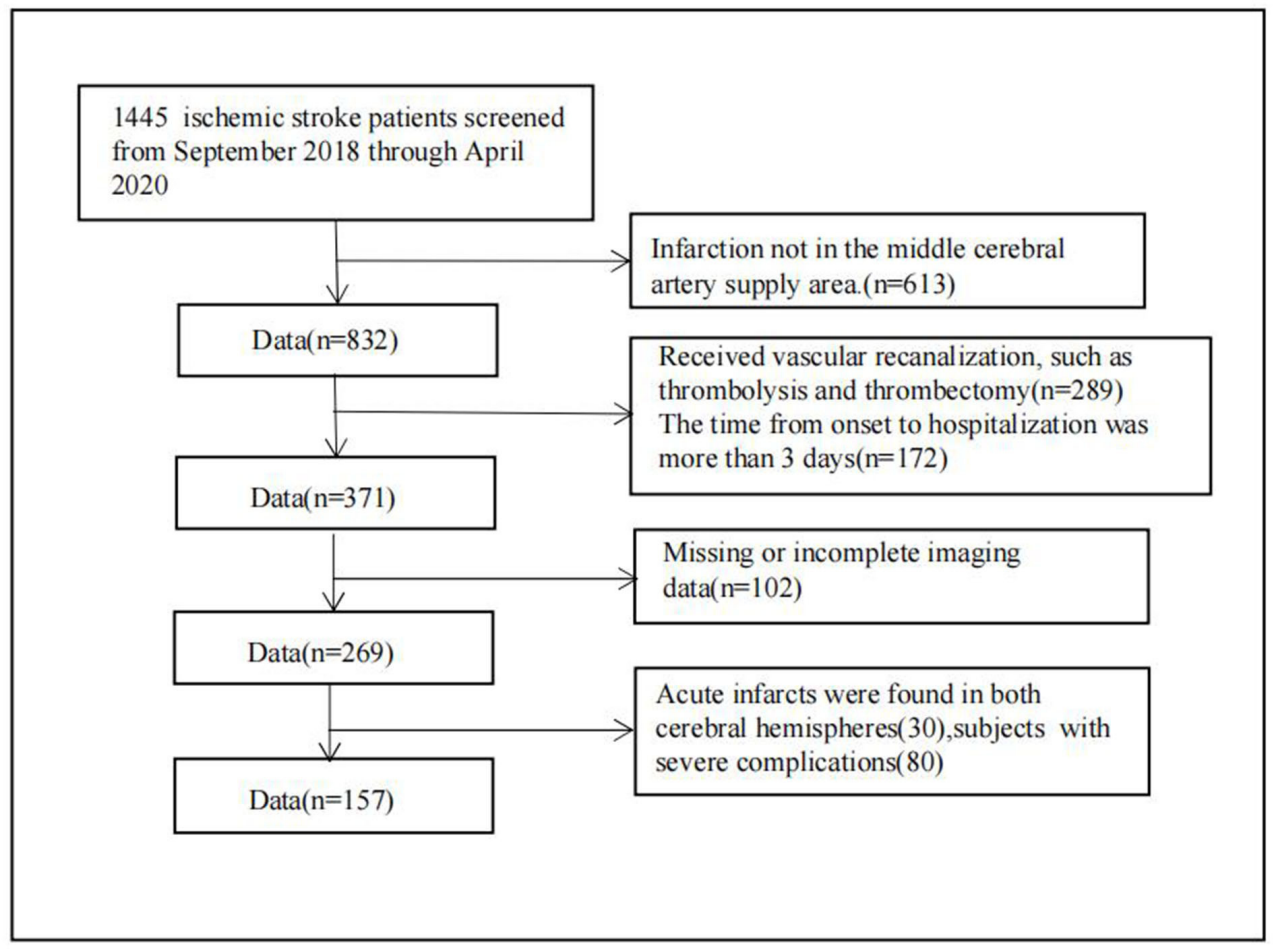
Flow chart of the systematic review screening process.

In this retrospective analysis, patients who experienced an ischemic stroke and were treated at Changzhou Second People's Hospital between September 2018 and April 2020 were considered for inclusion. Patients were enrolled if they met the following inclusion criteria (Figure 1): (1) age at the time of stroke onset was 18–80 years; (2) time from stroke onset to hospitalization was <3 days; (3) MRI, including diffusion-weighted imaging (DWI), SWAN, and MR angiography/computed tomography (CT) angiography, was performed within 7 days after stroke symptom onset; and (4) DWI indicated the presence of infarction in the middle cerebral artery supply area. Only patients with infarction in the middle cerebral supply area were included to avoid any differences caused by different locations of infarcted vessels. Exclusion criteria were as follows: (1) patients underwent vascular recanalization, such as thrombolysis or thrombectomy; (2) patients had severe artifacts on MRI; (3) patients had acute infarcts in both cerebral hemispheres; (4) patients also had severe cardiopulmonary insufficiency, renal insufficiency, and severe pneumonia.

### Assessment of APVS on SWAN

Patients underwent DWI, including SWAN, within 3 days of admission. All sequences were performed using 3T MRI (GE, Discovery MR 750). SWAN scanning parameters were as follows: repetition time (TR), 85.4 ms; echo time (TE), 44.65 ms; flip angle, 15; slice thickness, 3.6 mm; slice spacing, 1.8 mm; image columns, 512; image rows, 512. Post-processing was performed using self-contained Functool software to obtain the corrected phase diagram (phase) and the reconstructed SWAN minimum intensity projection (MIP) diagram. DWI scanning parameters were as follows: TR, 6,000 ms; TE, 79.5 ms; flip angle, 90; slice thickness, 5 mm; slice spacing, 6.5 mm; image columns, 256; image rows, 256.

Two senior neurologic physicians interpreted the MR images to assess APVS. The consistency of these interpretations was tested using the kappa coefficient, which was found to be 0.82. The evaluation of one of the physicians was then randomly selected for each case.

**Figure 2:**
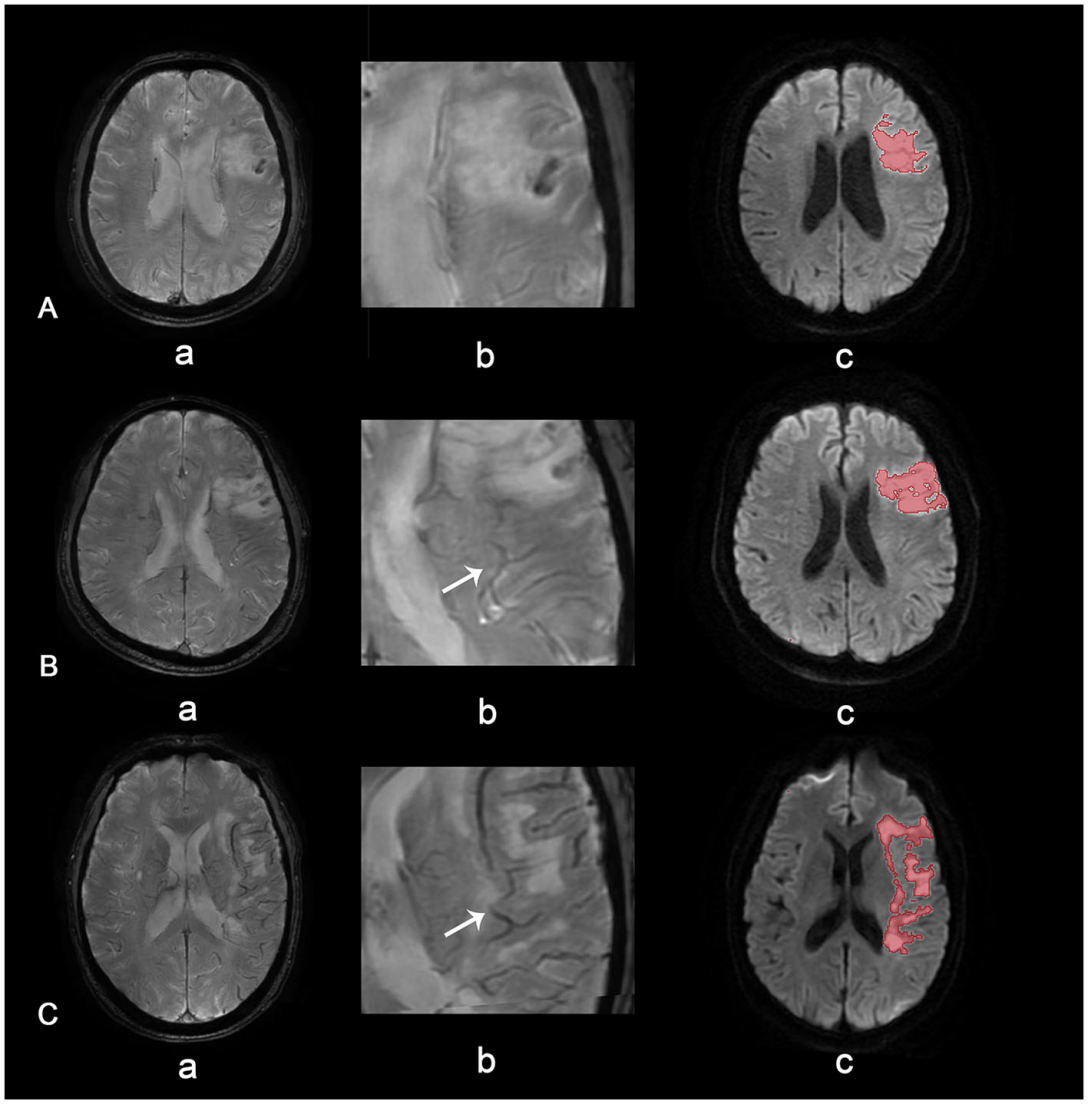
APVS grades. APVS grade 0 **(A)**, grade 1 **(B)**, and grade 2 **(C)**. Row (a) shows SWAN images of different grades, row (b) shows magnified APVS, and row (c) shows diffusion-weighted images with quantified volume.

Asymmetrical prominent veins sign is defined as an increased number of drainage veins and thickened diameter of veins (14). When the number and diameter of veins are roughly similar, the case is deemed APVS negative. Grades of APVS are defined as follows (Figure 2): in grade 2, veins on the affected side are significantly thicker and more plentiful than on the healthy side; in grade 1, veins on the affected side are slightly thicker and more plentiful than on the healthy side; and grade 0, there is no significant difference in veins between the affected side and the healthy side (15).

All MR images were interpreted by two senior neurologic physicians who were blinded to the patient's basic clinical information. When there was disagreement, the consensus was reached through discussion.

Although APVS can be subdivided into AMVS and ACVS depending on the location, we found that APVS was usually identified around the cerebral infarction tissue and was related to the location of cerebral infarction. Therefore, this study did not distinguish between AVMS and ACVS in patients with APVS.

The volume of cerebral infarction on DWI was measured using ITK-snap software (http://www.itksnap.org), and the distribution of cerebral veins was compared between the affected side and the healthy side. Furthermore, this study also measured the volume of white matter hyperintensities (WMH) using the W2MHS toolbox (https://www.nitrc.org/projects/w2mhs) on Matlab.

### Definition of END

Early neurological deterioration is usually defined as clinical deterioration or recurrence within 72 h after an ischemic stroke (16). For this study, END was defined as a National Institutes of Health Stroke Scale (NIHSS) score of more than 2 points recorded within 72 h after admission (17, 18). Previous studies have demonstrated that the occurrence of END is related to multiple factors such as blood pressure at admission, blood glucose level, NIHSS score, atrial fibrillation, and the presence of intracranial large vessel lesions (12, 13, 19). In this study, we assessed several other potential risk factors, including the occurrence of hemorrhagic transformation (HT) (based on criteria from the European Cooperative Acute Stroke Radiological Study) (20).

### Statistical analysis

The data were analyzed using IBM SPSS statistics 25 (IBM Corp.). Continuous data were assessed with the Kolmogorov-Smirnov normality test; results with normal distribution were expressed as mean ± SD, and those with non-normal distribution were expressed as median (25th−75th percentiles). Categorical variables were expressed as the number of cases (proportion). Differences between groups were analyzed using either independent samples *t*-tests, chi-square tests, or Mann–Whitney *U* tests. Crude binary regression was used to analyze the effect of APVS on END. In addition, multivariate binary regression was used for adjusting related factors considering the literature and statistical results in Table 1. APVS grades were included in the regression as categorical (*P*) and numerical (*P* for trend) variables. Spearman correlation was also used to assess the association between APVS grades and infarct volume, WMH volume, and offending vessel. *P*-values < 0.05 were considered to be significant.

**Table 1:** Clinical characteristics of patients with and without END.

	**Patients without END (*N* = 123)**	**Patients with END (*N* = 34)**	***P*-value**
Age, y, mean ± SD	61.00 ± 13.43	66.71 ± 13.51	0.030
Sex, *N* (%)	81 (65.9%)	28 (82.4%)	0.065
LDL, mmol/L	1.41 ± 0.70	1.51 ± 0.81	0.501
HCY, mmol/L	10.53 ± 3.86	11.97 ± 4.82	0.070
Hypertension, *N* (%)	92 (74.8%)	27 (79.4%)	0.578
Diabetes, *N* (%)	48 (39.0%)	14 (41.2%)	0.820
Smoke currently, *N* (%)	34 (27.6%)	14 (41.2%)	0.129
Atrial fibrillation, *N* (%)	1 7(13.8%)	7 (20.6%)	0.332
Admission NIHSS (IQR)	6 (3, 8)	5 (3, 7)	0.315
Offending vessel			0.051
M1	15 (12.2%)	8 (23.5%)	
M2	26 (21.1%)	11 (32.4%)	
M3-M4	82 (66.7%)	15 (44.1%)	
Infarct volume, mL (IQR)	22.95 (10.36, 41.96)	41.27 (22.99, 58.83)	<0.001
TOAST classification			0.031
Large vessel atherosclerosis	72 (58.5%%)	22 (64.7%)	
Cardioembolism	15 (12.2%%)	9 (26.5%)	
Small vessel occlusion	25 (20.3%%)	1 (2.9%)	
Uncertain and other cause	11 (8.9%%)	2 (5.9%)	
HT, *N* (%)	7 (5.7%)	6 (17.6%)	0.059^*^
Large artery stenosis	14 (11.4%)	9 (26.5%)	0.054^*^
WMH, mL (IQR)			
Periventricular	3.23 (0.90, 9.24)	8.03 (1.92, 11.83)	0.038
Deep	0.75 (0.27, 3.83)	3.53 (0.56, 7.59)	0.018
APVS (grades), *N* (%)			0.001
0	66 (53.7%)	7 (20.6%)	
1	43 (35.0%)	16 (47.1%)	
2	14 (11.4%)	11 (32.4%)	
APVS (location), *N* (%)	123	34	

LDL, low-density lipoprotein; HCY, homocysteine; NIHSS, The National Institutes of Health Stroke Scale; IQR, interquartile range; WMH, white matter hyperintensities; APVS, asymmetrical prominent veins sign; HT, hemorrhagic transformation; TOAST, Trial of Org 10172 in Acute Stroke Treatment.

Large artery stenosis refers to stenosis of large vessels including MCA M1-2 ≥70%.

* Continuity correction.

## Results

### Clinical characteristics of patients with END and those without END

Of the 157 enrolled patients (mean age, 62 years), 34 were diagnosed with END. As shown in Table 1, 27 of 84 patients with APVS were diagnosed with END, and 7 of 72 patients without APVS were diagnosed with END. Patients with END had higher grades of APVS than those without END (*P* < 0.05, *x*^2^ = 14.659). Patients with END were also older, had larger infarcts, and had higher WMH volumes than those without END (*P* < 0.05 for all). More particularly, patients with END had a higher periventricular WMH volume (*P* = 0.038) and a higher deep WMP volume (*P* = 0.018) than those without END. The groups also differed in etiological TOAST (Trial of Org 10172 in Acute Stroke Treatment) subtypes (*P* < 0.05). The associations between offending vessel and END (*P* = 0.051) and HT and END (*P* = 0.059) trended toward statistical significance.

### Risk factors for END

Binary regression analysis showed that APVS overall was related to END (*P* for trend <0.001, OR = 2.73, 95% CI, 1.59–4.69). APVS grade 1 and grade 2 were also found to be related to END (grade 1, *P* = 0.011, OR = 3.51, 95% CI, 1.33–9.23; grade 2, *P* < 0.001, OR = 7.41, 95% CI, 2.44–22.46). After adjusted age and sex, APVS remained an independent risk factor of END (*P* for trend <0.001, OR = 3.03, 95 CI%, 1.66–5.51).

Compared with APVS grade 0, the multivariable-adjusted OR for APVS grade 2 was 6.39, and 2.98 for grade 1 after adjusting age, sex, infarct volume, etiology of TOAST classification, and white matter hyperintensities. APVS remained an independent risk factor for END (*P* for trend = 0.003, OR = 2.56, 95% CI, 1.38–4.75) (Table 2).

**Table 2:** Risk factors for END.

**APVS**	**Crude**		**Multivariate model 1**		**Multivariate model 2**	
	**OR (95% CI)**	***P*-value**	**OR (95 CI%)**	***P*-value**	**OR (95% CI)**	***P*-value**
Grade						
0	1		1		1	
1	3.51 (1.33, 9.23)	0.011	3.24 (1.19, 8.80)	0.021	2.98 (1.06, 8.31)	0.038
2	7.41 (2.44, 22.46)	<0.001	9.08 (2.72, 30.37)	<0.001	6.39 (1.82, 22.34)	0.004
*P* for trend	2.73 (1.59, 4.69)	<0.001	3.03 (1.66, 5.51)	<0.001	2.56 (1.38, 4.75)	0.003

Crude: APVS grades enrolled in multivariate binary regression.

Multivariate model 1: Age and sex were adjusted.

Multivariate model 2: Age, sex, infarct volume, etiology of TOAST classification, and white matter hyperintensities were adjusted. APVS grades were enrolled in the regression as categorical (P) and numerical (P for trend) variables.

APVS, asymmetrical prominent veins sign; END, early neurological deterioration; TOAST, Trial of Org 10172 in Acute Stroke Treatment.

### Spearman correlation between APVS grades and potential risk factors

The APVS grades were positively correlated with infarct volume and 3-month modified Rankin Scale score (*r* = 0.289 and *r* = 0.203, respectively; *P* < 0.05 for both) and were negatively correlated with offending vessels (*r* = −0.17, *P* = 0.034) (Table 3).

**Table 3:** The Spearman correlation between APVS grade and risk factors.

	***R***	***P*-value**
Infarct volume (mL)	0.289	<0.001
Deep WMH	0.148	0.064
Periventricular WMH	0.140	0.080
Offending vessel	−0.170	0.034
3-month mRS score	0.203	0.011

WMH, white matter hyperintensities; APVS, asymmetrical prominent veins sign; mRS, modified Rankin Scale.

## Discussion

In this study, we found that 84 of 157 patients demonstrated APVS on MRI, and 34 of 157 patients were diagnosed with END. Analysis showed that the presence of APVS was strongly associated with the occurrence of END.

Early neurological deterioration is one of the main factors associated with poor prognosis in patients with acute ischemic stroke, and the incidence of END in these patients may be as high as 40% (21). Previous studies have found that stroke severity, development of cerebral edema, HT, age, hypertension, and diabetes are all related to the occurrence of END (13, 19, 22). Identifying such risk factors is crucial, as early treatment can help to prevent this serious complication.

When a stroke occurs, blood perfusion of brain tissue decreases, and the ischemic tissue absorbs the oxygen of residual blood. Low perfusion and vascular compensatory hemodynamic changes occur in local brain tissue, resulting in an imbalance between oxygen supply and demand in the ischemic area, an increase in oxygen uptake fraction, or an increase in expansion and volume of the drainage vein. This change may be due to venous enlargement after cerebral infarction as the infarct area attempts to obtain compensatory blood flow. Patients in the current study did not undergo vascular recanalization for various reasons. For some patients, the time window for thrombolysis or thrombectomy at the hospital had passed; for others, thrombolysis was not available for economic reasons or because there was a recent history of surgery. Among these patients who did not undergo thrombolytic therapy, the presence of APVS was found to be a risk factor for END. Previous studies using magnetically sensitive sequences found that in patients who had not yet undergone recanalization, the presence of APVS indicated recanalization, suggesting a favorable outcome (8, 9). In other studies of patients who did not undergo thrombolysis, the presence of APVS was found to be indicative of adverse outcomes (11, 23), similar to our findings in this study.

Asymmetrical prominent veins sign refers to asymmetry in the superficial cortical veins and deep medullary veins. Patients with APVS demonstrate an increased number of these veins or thickening of the diameter of these veins on the focal side of the cerebral hemisphere (7, 11). The appearance of APVS is the manifestation of the activation of metabolic reserve in the acute stage of ischemic stroke. When the cerebral perfusion pressure decreases, the ischemic brain tissue increases the oxygen extraction fraction to maintain the normal oxygen metabolism rate, thus increasing the concentration of deoxyhemoglobin in the drainage vein on the ischemic side. As a result, the drainage vein of the cerebral hemisphere in the corresponding region may demonstrate a prominent low signal on SWAN (9).

Susceptibility-weighted angiography is a technique that is based on the T_2_-weighted gradient-echo sequence in MRI. It is particularly sensitive to paramagnetic substances such as deoxyhemoglobin, hemosiderin, calcification, and iron deposition. It can not only provide important information for the diagnosis and treatment of neurodegenerative diseases but can also predict the bleeding transformation risk in patients treated with antithrombotic therapy through the detection of microbleeds (2, 24). In addition, studies have shown that cortical APVS demonstrated on SWAN/SWI can reflect the cerebral collateral circulation and cerebral hemodynamics and can be used to evaluate the compensatory state of patients with ischemic stroke from the perspective of blood flow and metabolism (10, 25).

The presence of APVS represents the occurrence of ischemia and hypoxia of brain tissue, and it indicates that collateral circulation is being established in the brain. The presence of APVS may therefore be a warning of irreversible damage to nerve function. If patients receive adequate reperfusion within the time window of thrombolysis or thrombolysis, it is very likely to save the ischemic penumbra, which may lead to a better prognosis. If patients do not receive reperfusion treatment, their disease is likely to worsen and lead to the development of END.

In this study, we found that patients with APVS tended to have a larger infarction volume than patients without APVS. If the infarct size is small, the venous deoxygenated hemoglobin is likely to be only slightly elevated, which results in the patient being negative for APVS. We also found that with the accumulation of WMH, the probability of APVS increases. Previous studies have shown that the APVS may be a factor that cannot be ignored in cerebral small vascular disease (26, 27). Researchers have also pointed out that dilation of the deep medullary vein and cortical vein sign plays a role in the other type of cerebral small vascular disease like microbleeds and white matter hyperintensities (28, 29).

This study had some limitations. First, APVS was rated visually despite the availability of recent literature regarding segmenting and quantifying veins. We also did not analyze dynamic changes in APVS after stroke; it remains unclear whether the APVS will persist or disappear in such patients. Additionally, the number of samples in this study was relatively small. Although the numbers were sufficient to carry out statistical analysis, our results should be interpreted with some caution because of the possibility of selection bias. Finally, in an attempt to obtain clinical consistency, we enrolled only patients with cerebral infarction of the middle cerebral artery; further studies assessing these factors in other arteries are needed.

## Conclusion

This study showed that APVS is closely related to END in patients who have experienced a stroke. In addition, we found that patients with a higher infarct volume are prone to demonstrate APVS on MRI.

## Data availability statement

The raw data supporting the conclusions of this article will be made available by the authors, without undue reservation.

## Author contributions

KH and JL participated in the design, data statistical analysis, and drafting of the manuscript. MZ participated in the design and helped with the data collection and analysis. WY helped with the data collection and analysis. YC helped to read and evaluate the retinal fundus photographs. KH and MZ were involved in the design, review, and editing of the manuscript and provided financial support. All authors contributed to the article and approved the submitted version.

## Funding

This project was supported by the National Natural Science Foundation of China (81671284), the General Program of Jiangsu Commission of Health (H2019051), and the Young Talent Development Plan of Changzhou Health Commission (CZQM2020073).

## Conflict of interest

The authors declare that the research was conducted in the absence of any commercial or financial relationships that could be construed as a potential conflict of interest.

## Publisher's note

All claims expressed in this article are solely those of the authors and do not necessarily represent those of their affiliated organizations, or those of the publisher, the editors and the reviewers. Any product that may be evaluated in this article, or claim that may be made by its manufacturer, is not guaranteed or endorsed by the publisher.
